# A homogenous nature of native Chinese duck matrilineal pool

**DOI:** 10.1186/1471-2148-8-298

**Published:** 2008-10-29

**Authors:** Da-Qian He, Qing Zhu, Shi-Yi Chen, Hui-Ying Wang, Yi-Ping Liu, Yong-Gang Yao

**Affiliations:** 1 College of Animal Science and Technology, Sichuan Agriculture University, Ya'an, Sichuan, PR China; 2 Institute of Animal Sciences, Shanghai Academy of Agricultural Sciences, Shanghai, PR China; 3 State Key Laboratory of Genetic Resource and Evolution, Kunming Institute of Zoology, Chinese Academy of Sciences, Kunming, Yunnan, PR China

## Abstract

**Background:**

China, with around 30 unique breeds, has a diverse duck genetic pool. Currently, there is no systematic report which investigates the genetic diversity, phylogenetic relationship, and matrilineal genetic structure of these domestic breeds and wild mallards (*Anas platyrhynchos*).

**Results:**

In this study, we sequenced the mitochondrial DNA (mtDNA) control region segments in 278 domestic ducks (*Anas platyrhynchos domestica*) from 19 indigenous breeds/populations and 70 wild mallard samples and analyzed them together with the 101 control region sequences from published sources. Fifty-two samples were then sequenced for a cytochrome *b *(Cyt *b*) gene fragment to solidify the pattern emerged from the control region sequences. All domestic duck and wild mallard haplotypes were essentially indistinguishable and were clustered together in the phylogenetic tree. There was no geographic differentiation and breed/population-specific distribution of duck lineages.

**Conclusion:**

Our results showed that unlike other domesticated farm animals in China such as chicken, cattle, goat, and yak with multiple matrilineal components, the matrilineal pool of Chinese ducks was homogenous.

## Background

Domestic ducks (*Anas platyrhynchos domestica*) play a key role in the agricultural and economic sectors of Asia. There are numerous domestic duck breeds in China including 27 indigenous breeds, two introduced breeds, and a few developing breeds according to the 2004 systematic field investigation [1]. About 70% of the recorded breeds are distributed along the mid to downstream regions of the Yangtze and Pearl rivers, as well as, in the coastal districts [1]. With the increasing demand for duck products, including meat, eggs, and down feathers, the duck breeding industry has flourished in China. In comparison to other domestic animals in China, the present conservation situation for local duck genetic resources is above average. Despite this, the overall population size of eight renowned breeds (Beijing duck, Youxian Sheldrake, Liancheng white duck, Jianchang duck, Jinding duck, Shaoxing duck, Putian black duck, and Gaoyou duck) has seen a sharp decline during the last decade. Fortunately, these local breeds are included in the National Genetic Resources Protection list http://www.agri.gov.cn/blgg/t20060609_626418.htm which ensures essential measurements are in place to sustainably manage these genetic resources.

The domestic duck is thought to have been domesticated independently from the wild mallard (*Anas platyrhynchos*), see a review by Li et al. [2]. A recent study of the mtDNA D-loop sequence variation among nine duck breeds along the Yangtze-Huai River also suggested a single matrilineal origin from the wild mallard (*Anas platyrhynchos*) [3]. However, comparisons of mtDNA polymorphisms among two local domestic ducks, the wild mallard and one wild spot-billed duck (*Anas zonorhyncha*), using RFLP (restriction fragment length polymorphism) showed that both wild duck species contributed to the gene pool of domestic ducks in Fujian Province, Southeast China [4]. Analysis of the nuclear DNA sequence variation in representative Chinese duck breeds, wild mallard, and spot-billed duck by RAPD (random-amplified polymorphic DNA) [5], AFLP (amplified fragment length polymorphism) [6] and SSR (simple sequence repeat, microsatellite) markers [7] further supported this notion. Based on the archaeological assemblages, Xie proposed that Chinese native ducks were domesticated in the middle and lower region of the Yangtze River more than 2,000 years ago [8]. After the initial domestication in China, the Chinese domestic ducks were subsequently introduced into Japan via Taiwan [9].

Overall, molecular studies investigating the duck breeds and populations in China are limited, focusing primarily on blood protein polymorphisms [10-12], random amplified polymorphic DNA [5,13], and microsatellite markers [7,14-17]. Much of the research is overshadowed by the superficiality of the research work (especially those published in Chinese) and the limited breeds and populations investigated. mtDNA sequence variation has been used extensively to study the genetic structure and matrilineal origin of farm animals [18-28]. However, mtDNA reports about domestic duck and wild mallard are relatively sparse [3,9,29,30]. In this study, we analysed the genetic diversity and phylogeographic profiles of 374 specimens from 26 Chinese domestic duck breeds/populations and five Thailand native duck samples based on mtDNA sequence variation. To better understand the matrilineal origin of domestic ducks in China, we also sequenced 70 individuals from three wild mallard populations and compared them with the domestic samples. Our results revealed that the Chinese native duck gene pool is homogeneous, without clear geographic differentiation of the regional breed/population pools.

## Results

### mtDNA control region and Cyt *b *sequence variation

**Figure 1:**
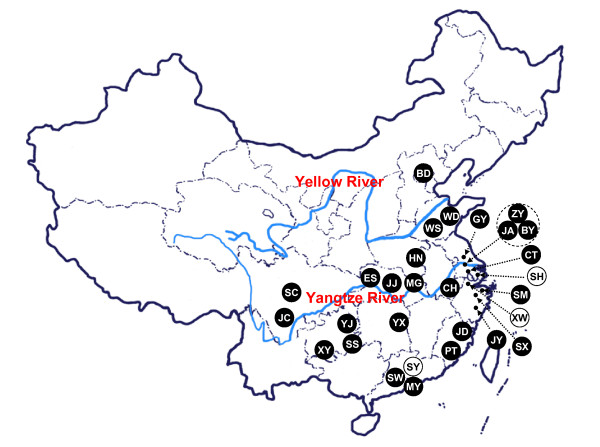
**Sample locations in this study**. Samples marked in black and white circles refer to the domestic ducks and the wild mallards, respectively. The abbreviations of different breeds/populations were defined in Table 1.

A total of 449 domestic duck and wild mallard samples were analyzed in this study (Table 1 and Figure 1). Among these samples, 374 individuals were from 26 Chinese domestic duck breeds/populations (including 96 reported sequences, which were shared by 22 haplotypes with GenBank accession numbers EF126702–EF126706, EF126708, EF126711–EF126718, EF126721, EF126723–EF126728, EF126731), five domestic duck samples (*Anas platyrhynchos*) were from Thailand (GenBank accession nos. EU013948–EU013952), and 70 individuals were from three wild mallard populations in China. Forty-one haplotypes were identified and were defined by 30 polymorphic sites in the 481 bp fragment (Additional file 1). Among these haplotypes, H6 was predominant and occurred in 282 samples from all 30 breeds/populations; haplotypes H22, H1, and H5 were distributed in 32, 21, and 12 individuals, respectively. Nineteen haplotypes were shared by two to ten samples, while the remaining eighteen haplotypes were found in only a single individual. The five Thailand native ducks shared the most common haplotype H6. Haplotype H14, a one-step mutation derive of haplotype H6, was only found in three wild mallard samples, whereas the other wild mallard samples shared haplotypes with the domestic samples. The detailed haplotype distribution pattern among different breeds/populations is shown in Additional file 2.

**Table 1:** Sample information and genetic diversity of Chinese domestic ducks and mallards

ID^a^	Breed/Population (Abbreviation)^b^	No.^c^	Location	Reference	Haplotype diversity (*h *± SD)	Nucleotide diversity (π ± SD)
01	Beijing duck (BD)	15 (1)	Beijing	This study	0.752 ± 0.076	0.00444 ± 0.00053
02	Weishan Sheldrake (WS)	26 (4)	Weishan, Shandong	This study; [3]	0.523 ± 0.116	0.00202 ± 0.00061
03	Wendeng black duck (WD)	7	Wendeng, Shandong	[3]	0.286 ± 0.196	0.00119 ± 0.00082
04	Shaoxing duck (SX)	30 (5)	Shaoxing, Zhejiang	This study	0.503 ± 0.106	0.00129 ± 0.00033
05	Jinyun Sheldrake (JY)	15 (1)	Jinyun, Zhejiang	This study	0.362 ± 0.145	0.00107 ± 0.00052
06	Cherry Valley duck (SM)	15 (2)	Zhenhai, Zhejiang	This study	0.562 ± 0.095	0.00406 ± 0.00070
07	Gaoyou duck (GY)	22 (2)	Gaoyou, Jiangsu	This study; [3]	0.537 ± 0.123	0.00142 ± 0.00040
08	CT (CT)	15 (2)	Jianhu, Jiangsu	This study	0.000 ± 0.000	0.00000 ± 0.00000
09	Ji'an Red duck (JA)	7 (1)	Zhenjiang, Jiangsu	This study	0.286 ± 0.196	0.00059 ± 0.00041
10	Zhenyi duck (ZY)	9 (3)	Zhenjiang, Jiangsu	This study	0.556 ± 0.165	0.00127 ± 0.00044
11	BY (BY)	7 (1)	Zhenjiang, Jiangsu	This study	0.000 ± 0.000	0.00000 ± 0.00000
12	Jinding duck (JD)	25 (4)	Fuzhou, Fujian	This study	0.363 ± 0.120	0.00082 ± 0.00030
13	Putian Black duck (PT)	14	Putian, Fujian	This study	0.582 ± 0.092	0.00133 ± 0.00028
14	Sanshui White duck (SW)	14 (2)	Sanshui, Guangdong	This study	0.725 ± 0.104	0.00380 ± 0.00079
15	Guangdong Sheldrake (MY)	16 (6)	Guangzhou, Guangdong	This study	0.600 ± 0.127	0.00187 ± 0.00056
16	Caohu Sheldrake (CH)	12	Lujiang, Anhui	[3]	0.576 ± 0.163	0.00167 ± 0.00060
17	Huainan Sheldrake (HN)	11	Gushi, Henan	[3]	0.836 ± 0.080	0.00280 ± 0.00051
18	Mianyang Sheldrake (MG)	12	Xiantao, Hubei	[3]	0.773 ± 0.128	0.00287 ± 0.00070
19	Jingjiang Sheldrake (JJ)	11	Danyang, Hubei	[3]	0.836 ± 0.070	0.00325 ± 0.00046
20	Enshi Sheldrake (ES)	11	Lichuan, Hubei	[3]	0.836 ± 0.089	0.00287 ± 0.00056
21	Youxian Sheldrake (YX)	9	Youxian, Hunan	[3]	0.806 ± 0.120	0.00381 ± 0.00098
22	Yinjiang duck (YJ)	15 (1)	Yinjiang, Guizhou	This study	0.562 ± 0.143	0.00135 ± 0.00042
23	Xingyi Duck (XY)	15 (1)	Xingyi, Guizhou	This study	0.371 ± 0.153	0.00135 ± 0.00070
24	Sansui duck	15	Sansui, Guizhou	This study	0.257 ± 0.142	0.00055 ± 0.00032
25	Sichuan Sheldrake (SC)	15 (1)	Ya'an, Sichuan	This study	0.562 ± 0.143	0.00135 ± 0.00042
26	Jianchang duck (JC)	11 (5)	Xichang, Sichuan	This study	0.800 ± 0.114	0.00325 ± 0.00076
27	Thailand native duck ((TH)	5	---	EU013948–EU013952^d^	0.000 ± 0.000	0.00000 ± 0.00000
***Subtotal***	---	***379 (42)***	---	---	***0.619 ± 0.029***	***0.00218 ± 0.00016***
28	Guangdong wild duck (SY)	14 (2)	Guangzhou, Guangdong	This study	0.143 ± 0.119	0.00030 ± 0.00025
29	The West Lake wild duck (XW)	31 (2)	Jiaxing, Zhejiang	This study	0.424 ± 0.095	0.00228 ± 0.00056
30	Shanghai wild duck (SH)	25 (6)	Fengxian, Shanghai	This study	0.633 ± 0.104	0.00215 ± 0.00052
***Subtotal***	---	***70 (10)***	---	---	***0.461 ± 0.072***	***0.00190 ± 0.00036***

**Total**	---	**449 (52)**	---	---	**0.598 ± 0.027**	**0.00217 ± 0.00015**

^a^Sample ID numbers 1–27 refer to the domestic duck breeds/populations; sample ID numbers 28–30 refer to the mallard populations.

^b^Abbreviation "CT" denotes the crossbred population of the Cherry Valley duck and the Gaoyou duck; "BY" represents the crossbred population of the Zhenyi duck and the Gaoyou duck.

^c^Numbers in the bracket refer to the number of samples that were sequenced for Cyt *b *fragment.

^d ^The five Thailand native domestic duck mtDNA sequences were deposited in GenBank by Leekaew P, Songserm T, Choothesa A, and Boonyaprakob U on July 3, 2007.

Based on the haplotype distribution pattern of the mtDNA control region sequences, we further selected 52 samples and analyzed a Cyt *b *fragment (Table 1), to solidify the pattern emerged from the control region sequences. Three haplotypes (defined by two variable sites 15274 A>T and 15574 A>G) were discerned in 52 Cyt *b *sequences analyzed in this study. Specifically, variant A15274T was detected in four Jianchang ducks and one Jinding duck and caused an amino acid change from Leucine to Phenylalanine. Variant A15574G was synonymous and appeared in one Jinding duck.

### Phylogenetic profile of domestic ducks

**Figure 2:**
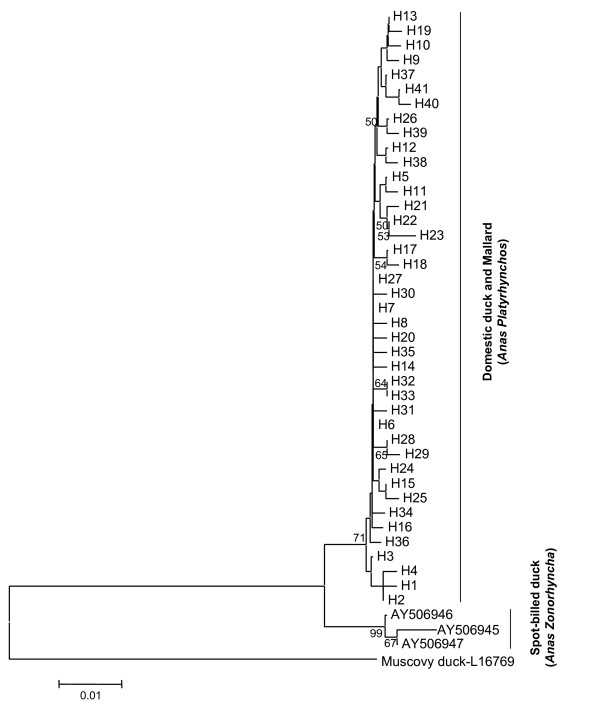
**Rooted neighbour-joining tree of Chinese domestic ducks and mallards based on the mtDNA control region haplotypes**. The tree was rooted by the Muscovy duck (*Cairina moschata*). The values on the branch are bootstrap support based on 1000 replications and those values lower than 50% were omitted.

**Figure 3:**
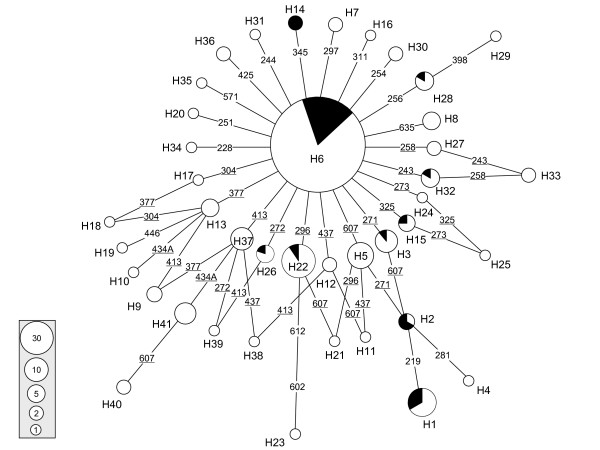
**Network profile of the mtDNA control region haplotypes in Chinese domestic ducks and mallards**. The links are labelled by the nucleotide positions to designate transitions; the single transversion at site 434 are further specified by adding suffix A. Recurrent mutations are underlined. The order of the mutations on a branch is arbitrary. The black colour denotes the wild mallards, while the white represents the domestic ducks. Circle area is proportional to haplotype frequency.

In the rooted neighbour-joining (NJ) tree based on the mtDNA control region haplotypes, all domestic duck and wild mallard samples were clustered together, whereas the spot-billed ducks formed another cluster (Figure 2). The wild mallard samples were intermingled with the domestic samples. There was no breed/population-specific clustering pattern. The network based on the mtDNA control region haplotypes revealed similar pattern as the NJ tree, with no essential distinction between the wild and domestic samples (Figure 3). Moreover, the network presented a star-like profile, consistent with a pattern of population expansion in the past. Twenty-three haplotypes differed from haplotype H6 by one mutation, and the remaining 17 haplotypes diverged from H6 by no more than three-mutation distance (Figure 3). The 70 wild mallard samples shared the predominant haplotype and eight other haplotypes with the domestic samples. The paucity of sequence variation in the Cyt *b *sequences was consistent with the less resolved tree based on the mtDNA control region sequences. We did not perform phylogenetic analysis for Cyt *b *sequences due to the lack of sequence variation.

### Genetic diversity of duck breeds

Among the 30 duck breeds/populations analyzed in this study, the genetic diversity of each population/breed varied substantially (Table 1). Only one haplotype was detected in each sample of the crossbred populations of the Cherry Valley duck, the Gaoyou duck (CT), the Zhenyi duck and the Gaoyou duck (BY), as well as, the Thailand domestic duck (TH). The Jingjiang Sheldrake (JJ) had the highest haplotype diversity, while the Beijing duck (BD) had the highest nucleotide diversity. The haplotype diversity and nucleotide diversity in all the domestic ducks was 0.619 ± 0.029 and 0.00218 ± 0.00016, respectively, which was higher than those of the wild mallard (haplotype diversity, 0.461 ± 0.072; nucleotide diversity, 0.00190 ± 0.00036). The average haplotype diversity and nucleotide diversity in all 449 samples was 0.598 ± 0.027 and 0.00217 ± 0.00015, respectively. The small sample size of some breeds/populations and their unique breeding histories may account for the high variability of genetic diversity (Table 1). In addition, potentially biased sampling, e.g. from one cultured population that is hard to avoid the potentially close affinity of individuals, could reduce the haplotype and nucleotide diversities.

### Population demographic history

**Figure 4:**
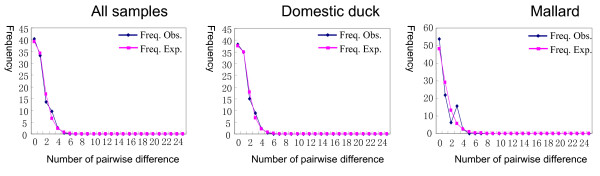
Mismatch distributions for Chinese domestic ducks and mallards.

We performed the *Fs *test of Fu [31] and the mismatch distribution analysis [32] to estimate the demographic history of duck. A statistically significant rejection of the neutrality hypothesis revealed by the *Fs *test [31] and a unimodal or Poisson-like mismatch distribution [32] would characterize a population expansion in the past. In contrast, a population with a constant size in the past would have a multimodal mismatch distribution and insignificant *Fs *test result [31,32]. As shown in Table 2, the *Fs *tests [31] for the domestic duck samples, wild mallard samples, and the total samples were all statistically significant (*P *< 0.05). The mismatch distributions for all samples (including domestic ducks and wild mallards) showed a (biased) unimodal shape (Figure 4). Similar distribution shape was detected for the domestic ducks. However, the wild mallard samples showed a seemingly bumpy mismatch distribution, which was characteristic of a population in equilibrium [32] and was consistent with an explanation for population fragmentation if it was not caused by the small sample size [33] or other demographic effects. Combined with the star-like shape of the network profile (Figure 3), we speculated that the domestic duck in China might have undergone population expansion in the past.

**Table 2:** Fu's *Fs *test for domestic duck and mallard samples

	Domestic duck (N = 379)	Mallard (N = 70)	Total (N = 449)
*F*_*S *_value	-52.761	-5.187	-54.022
*P*-value	0.000	0.004	0.000

Note: Values were estimated based on the mtDNA control region sequences.

**Figure 5:**
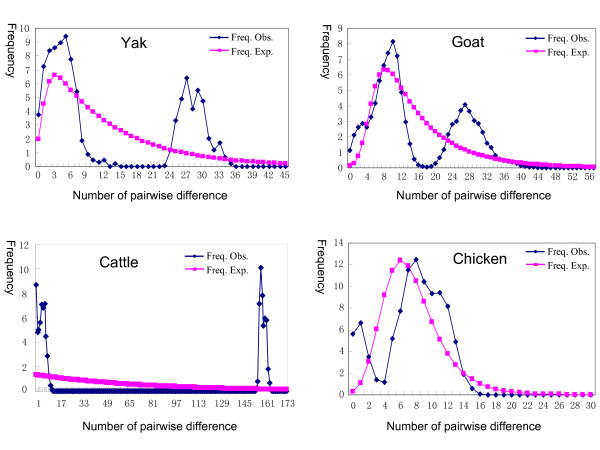
**Mismatch distributions for domestic yak, goat, cattle, and chicken in China**. The data were from our previous studies and others [21-23,39]. The sample sizes for yak, goat, cattle, and chicken were 52, 795, 209, and 900, respectively.

To further compare the results with other farm animals in China, we estimated the mismatch distributions for animals, such as domestic yak, goat, cattle, and chicken (Figure 5). All these farm animals showed multimodal mismatch distribution patterns, especially yak, goat, and cattle, which were apparently caused by multiple divergent matrilineal components in each pool (Table 3), instead of indicating a population in equilibrium. Indeed, the multimodal mismatch distributions of Chinese cattle were derived from the existence of both *Bos taurus *and *Bos indicus *types [22,34]. An analysis of the mismatch distributions based on *Bos taurus *or *Bos indicus *types only showed a unimodal distribution (data not shown). Overall, the mismatch distribution patterns of goat, yak, cattle, and chicken were dramatically different when compared to that of the domestic duck. Note that the comparison between the domestic duck and other farm animals was restricted to their current matrilineal genetic structure and the domestication history of each animal was not considered.

**Table 3:** Main matrilineal components in Chinese domestic animals

Animal	No. of haplogroups^a^	Reference
Chicken	Seven (A-G)	[21]
Cattle	Six (T1–T4, I1–I2)	[22,34]
Water buffalo	Two (A-B)	[28]
Yak	Six (A-F)	[23,27]
Goat	Four (A-D)	[26,39]
Pig	Four (D1–D4)	[18]
Donkey	Two (A-B)	[48]

^a^We did not consider the nested sub-haplogroups within each main haplogroup and neglected the potentially different age estimates for each haplogroup when counting the number of haplogorups.

## Discussion

In this study we discerned the genetic diversity and origin of Chinese native ducks. The weak phylogeographic structure of Chinese domestic ducks and wild mallards is clearly discerned in the NJ tree and the network profile based on the mtDNA control region haplotypes. The results suggest that the matrilineal pool of Chinese domestic ducks is homogeneous and all samples can be grouped into one haplogroup, which is analogous to a single matrilineal origin. The Cyt *b *gene sequences in 52 domestic duck and wild mallard samples that were selected based on their control region sequence information contain only two variable sites, which further supports the homogeneous nature of the duck matrilineal pool. The overall diversity of Chinese domestic duck estimated in our study was similar to the recent reports based on the microsatellite markers [14] and the mtDNA control region sequence variation [3].

In a recent study, Hitosugi and colleagues analyzed the Cyt *b *sequence variation of duck samples from East and Southeast Asia, and they found that the samples from Taiwan and Japan were grouped into the Northeast Asian group, whereas those from Indonesia, Vietnam, and west Malaysia were clustered into the Southeast Asian group [9]. This paraphyletic pattern of duck mtDNAs directly contrasts the clustering pattern of Chinese samples. Because we analyzed up to 26 domestic duck breeds/populations (which covers about 80% of the recorded native duck breeds/populations in nearly all current habitats across China; Table 1 and Figure 1) and three wild mallard populations, we believed that our sampling could represent the entire matrilineal pool of Chinese duck (at least for the Mainland region) and we should identify lineages belonging to the two groups. An audit for the Cyt *b *sequences that were grouped into the Southeast Asian group reported by Hitosugi et al. [9] showed that all these sequences should be nuclear mitochondrial pseudogenes (NUMTs), as these Cyt *b *fragments contained a premature termination codon. The invasion of NUMT is not infrequent in birds [35-37] and it had been identified in several goose species [36]. Among the two haplotypes in the Northeast Asian group [9], one shared sequence with the duck reference sequence (GenBank accession number NC_009684) and the other was a close derivative of the main haplotype in Chinese duck. Therefore, after eliminating the NUMTs in the data of Hitosugi et al. [9], the emerging pattern from their duck Cyt *b *sequences is consistent with our results. In fact, Hitosugi et al. [9] also sequenced the mtDNA control region sequences for these East Asian duck samples and observed no variation; a result that concurs with our findings.

An important issue which needs clarification concerns our belief that the mtDNA sequence data generated in the present study were free of NUMTs and DNA contamination, although we did not perform specific experiments for confirmation. Three lines of evidence justify our claim. First, the mutation spectrum left by spurious amplification of a NUMT could be detected because it usually is considerably different from the authentic natural spectrum [38]. Among all blood/tissue samples analyzed in this study, we did not detect any sequences showing a remarkable divergence (which would be resulted from the different evolutionary rate between authentic mtDNA and the NUMTs). Second, we obtained consistent patterns between two different mtDNA fragments and all Cyt *b *sequences could be fully translated. Third, DNA contamination between different samples and co-amplification of NUMTs and authentic mtDNA would result in many heteroplasmic mutations in the sequence, which was not observed in this study.

Interestingly, the matrilineal pool homogeneity of Chinese native ducks differed substantially with other domesticated Chinese animals, as judged by the numbers of main matrilineal components (Table 3) and the mismatch distribution patterns (Figures 4 and 5). In contrast to the unimodal shape for Chinese native ducks, the mismatch distributions for Chinese domestic yak [23,27], goat [26,39], cattle [22,25,34], and chicken [20,21] were bumpy or multimodal, which reflects the highly divergent lineages within the population (Table 3). Multiple domestication events of these latter farm animals could account for the difference in their respective matrilineal pools. We did not attempt to discern the domestication time for Chinese domestic ducks in this study because of concerns with the molecular clock [40-42]. Similarly, the indistinguishable matrilineal components among different breeds/regional populations of both domestic ducks and wild mallards have deterred any attempts to elucidate the initial domestication site(s) of Chinese domestic ducks.

Kulikova and colleagues detected two mtDNA haplogroups (A and B) of mallards that were collected from western Russia to mainland Alaska, and found that the haplogroup A was predominantly distributed in western Russia and North Asia territories [29,30]. All Chinese domestic duck and wild mallard mtDNAs in this study could be grouped into haplogroup A, suggesting a close relationship between the mallard matrilineal pools of China and North Asia. The fact that five Thailand domestic duck samples shared haplotype H6 with Chinese domestic ducks and wild mallards is also evidence for a genetic continuality among these regional pools. Nonetheless, due to the small sample size of the Thailand domestic duck, such a speculation should be interpreted with caution.

During the past decade, many molecular studies (mainly based on nuclear genetic markers) showed that the wild mallard and spot-billed duck have contributed to the genetic pool of Chinese domestic ducks, with the mallard contributing a greater proportion [1,4-7]. However, in the phylogenetic tree based on the mtDNA control region sequences, the three spot-billed ducks were robustly separated and none of the Chinese domestic ducks and wild mallards (including the Thailand sample) were clustered with the spot-billed ducks. A possible explanation for this unique pattern is that the genetic contribution of spot-billed duck to Chinese domestic duck pool might be via a male-biased gene introgression, as have been described between the eastern spot-billed duck and the mallard [29]. Study of the paternal markers in wild spot-billed duck, mallard, and domestic ducks will help to clarify this important issue.

## Conclusion

In this study, we analyzed Chinese domestic duck and wild mallard mtDNA control regions and partial Cyt *b *sequences variation. The results showed that all domestic duck and wild mallard haplotypes were essentially indistinguishable and were clustered together in the phylogenetic tree. The matrilineal pool of Chinese domestic ducks and mallards was rather homogeneous, without clear geographic differentiation of the regional breed/population pools. This pattern substantially differs from other farm animals in China.

## Materials and methods

### Sampling, DNA amplification and sequencing

In total, we collected 348 blood/tissue samples from 19 Chinese domestic duck breeds/populations (N = 278) and three wild mallard populations (N = 70) in East, Central and South China (Table 1 and Figure 1).

Genomic DNA was extracted from blood/tissue samples using a standard phenol/chloroform method. The mtDNA control region fragments (481 bp) were amplified and sequenced using primer pair L194: 5'-CCTACCTATCGGACTACCCTC-3'/H716: 5'-GCAGGTGTGTCCAGGCTTAGA-3', which was designed in this study. PCR amplification was performed in a 50-μl reaction mixture containing 100 ng of DNA, 10 mM Tris – HCl (pH 8.3), 2.5 mM MgCl_2_, 50 mM KCl, 10 pM of each primer, and 1 unit of *Taq *polymerase following 33 cycles of 50 s at 94°C, 40 s at 58°C, and 90 s at 72°C. PCR products were purified on spin columns and were directly sequenced for both strands using Big Dye Terminator v3.1 Cycle Sequencing Kit (Applied Biosystems, USA) on an ABI PRISM^® ^3100 DNA sequencer according to the manufacturer's manual.

The Cyt *b *fragment (508 bp) was amplified and sequenced using primer pair designed in this study (L15230: 5'-ACCCTGACCCGATTCTTC-3'/H15774: 5'-GATGCGAGTTGCCCGATGA-3') under the same condition as for mtDNA control region analysis but with a modification of annealing temperature (55°C) during the PCR amplification.

### Data analysis

The domestic duck and wild mallard mtDNA control region and Cyt *b *sequences were edited and aligned using the DNAstar program (DNAS Inc, Madison, WI, USA). The sequences obtained were aligned based on the complete mitochondrial genome of *Anas platyrhynchos *(GenBank accession number NC_009684). We retrieved 106 Chinese domestic duck mtDNA control region sequences from nine native breeds/populations (including two breeds that were overlapped with our sampling) and five Thailand native ducks (GenBank accession numbers EU013948–EU013952) from GenBank. All these reported control region sequences were aligned with the new sequences generated in this study and were truncated to 481 bp fragments in the following analyses. Ten reported mtDNA sequences (shared by nine haplotypes; GenBank accession numbers EF126707, EF126709, EF126710, EF126719, EF126720, EF126722, EF126729, EF126730, and EF126732) were excluded for further analysis because of apparent sequencing and reading errors by applying an error-pinpointing approach described in our previous study and others [43,44]. Three mtDNA control region sequences of the spot-billed duck (*Anas zonorhyncha*) were retrieved from GenBank (accession numbers AY506945–AY506947) and were included in the NJ tree analysis to discern their phylogenetic relationship to domestic samples.

The NJ tree was constructed based on the haplotypes identified in domestic ducks, mallards, and spot-billed ducks and the Kimura-2-parameters model using MEGA 4.0 [45]. The Muscovy duck (*Cairina moschata*; GenBank accession number L16769) was chosen as the outgroup to root the tree. Median-joining network of the mtDNA control region sequence haplotypes was constructed according to Bandelt et al. [46] using program Network 4.1 http://www.fluxus-engineering.com/sharenet.htm. We estimated the haplotype diversity (h) and nucleotide diversity (π) of the breeds/populations using DnaSP 4.10 [47]. The *Fs *values of the neutrality test [31] and the mismatch distributions [32] for the domestic ducks and wild mallards, as well as the total samples were analyzed using DnaSP 4.10 [47]. We also estimated the mismatch distributions for some reported Chinese domestic animals, such as chicken, cattle, yak, and goat [21-23,39], in comparison to that of the duck. The new duck mtDNA control region and Cyt *b *sequences generated in this study were deposited in GenBank under accession numbers EU677846–EU678193 and EU678194–EU678245, respectively.

## Authors' contributions

DQH collected samples, carried out the molecular genetic studies, participated in the sequence alignment, analyzed the data, and wrote the manuscript. QZ participated in the design of the study and helped to draft the manuscript. SYC collected the samples, carried out the molecular genetic studies, participated in the sequence alignment, analyzed the data, and wrote the manuscript. HYW collected samples. YPL conceived of the study, and participated in its design and coordination and wrote the manuscript. YGY conceived of the study, supervised the data analyses and interpretation, and wrote the manuscript. All authors read and approved the final manuscript.

## Supplementary Material

Additional file 1 Sequence variation of 41 mtDNA control region haplotypes identified in 449 domestic ducks and wild mallards. Variable sites were scored relative to the reference sequence (abbreviated as RS, GenBank accession number NC_009684). Dots (·) denote identity with the reference sequence, and the number of samples sharing the same haplotype is listed in the right column (under the capital N). Click here for file

Additional file 2 Haplotype distribution among different domestic duck breeds/populations and wild mallard populations. Click here for file
