# Supplementary material for: A homogenous nature of native Chinese duck matrilineal pool
Source: BMC Evol Biol. 2008 Oct 29;8:298. doi: 10.1186/1471-2148-8-298 (PMC2586638; doi:10.1186/1471-2148-8-298)
Supplement: Additional file 1 — Sequence variation of 41 mtDNA control region haplotypes identified in 449 domestic ducks and wild mallards. Variable sites were scored relative to the reference sequence (abbreviated as RS, GenBank accession number NC_009684). Dots (·) denote identity with the reference sequence, and the number of samples sharing the same haplotype is listed in the right column (under the capital N). [file 1471-2148-8-298-S1.doc]

**Additional file 1**

Sequence variation of 41 mtDNA control region haplotypes identified in 449 domestic ducks and wild mallards. Variable sites were scored relative to the reference sequence (abbreviated as RS, GenBank accession number NC_009684). Dots (·) denote identity with the reference sequence, and the number of samples sharing the same haplotype is listed in the right column (under the capital N).
